# Health Resorts and Outing Places

**Published:** 1919-04

**Authors:** 


### Health Resorts and Outing Places.

Physicians will be interested in the May issue of The Texas Medical Journal which will be a special Health Resort and Outing number. Doctors are recognizing the fact that it is essential for people to get away from home some time during the year to rest and recuperate if they would stay well. Of course, the sick are being sent constantly to the health hesorts of the country that cater to the special disease with which they are suffering, but people who are well now consult their physicians as to the best places to go to in order that they may get the most benefit from their vacations. That is why the May number will be given largely to help doctors know something of such place. In the South most people plan their health vacations for the summer months.

